# New Appliances and Things Medical

**Published:** 1899-08-19

**Authors:** 


### NEW APPLIANCES AND THINGS MEDICAL.

[We shall be glad to receive, at our Office, 28 & 29, Southampton Street, Strand, London, W.C., from the manufacturers, specimens of all new preparations and appliances which may be brought out from time to time.]

### OLD COGNAC BRANDY.

(Edward Archer and Co., Gt. Malvern.)

We have examined a sample of the brandy supplied by this firm. It appears a well-matured spirit with an alcoholic percentage of about 47·0 (by volume). It has the aromatic odour such as is peculiar to a genuine product of Cognac, and from our observation we are prepared to recommend it strongly for invalid use. By such as have to take brandy medicinally it will be found not only highly fragrant and agreeable, and with the rapid diffusibility of a pure spirit its stimulating properties will be immediately appreciated.

### I. AND I. FOOD FOR INVALIDS AND INFANTS.

(The Infant and Invalid Food Company, Cereal Buildings, 54, City Road, London.)

This new prepared food is one of considerable concentration, and contains in an exceedingly assimilable form all the elements that are necessary to build up or maintain the human frame. It contains a high percentage of fat. For nursing mothers and invalids it should be of considerable value, and also for growing children. As is true of all such prepared foods, it may be of value also for infants under certain conditions, but it should not be substituted for cows' milk, or added to it under normal conditions, unless, indeed, under medical supervision, and with the full knowledge that it is so easy of digestion that no encouragement is given by it to the natural functions of the stomach.

### VERMITE INSECT POWDER AND BLOWER.

(John Calabrese and Co., Limited, 26, Billiter Buildings, Billiter Street, London, E.C.)

The insect powder prepared by this firm has certainly an excellent effect as a vermin destroyer; black beetles show great intolerance of its action, and for moths, ants, and other pests of a similar nature it is equally efficacious. The householder, and more particularly the cook, will find it of the greatest utility in exterminating any such undesirable inhabitants of the basement or cupboards. The blower in which the powder is now most ingeniously sold consists of a modified insufflator, something of the nature of ordinary bellows; the long nozzle through which the insect powder is blown can be inserted between boards or into chinks or cracks in the walls, through which the beetle or other pests are known to make their egress. It is a practical and really useful invention.

### THE ERENA BED CRUTCH.

This little invention, which is the outcome of a nurse's ingenuity, strikes us as being likely to be of great use and comfort to such invalids and paralytics as are confined to their beds and require some artificial support to maintain the sitting posture. The crutch can be of any desired length, fits comfortably under the arm, and, being rounded off at the lower end, can obtain a good foothold in the bedding. The crutch can be obtained of F. Schultze and Co., 89, Southwark Street, Blackfriars, S.E.

### LEAD SEALS.

(Marshall and Co., Belsize Works, Clayton, Manchester.)

Lead seals have been extensively used by merchants who send out their goods in sacks, as they have found them a most convenient means of sealing the string with which they tie up the necks and in such a way that they cannot be tampered with. Messrs. Marshall are the possessors of patents which allow the manufacture of these seals to be effected at avery low price, and they can be supplied in all sizes with special pincers for impressing any required design or mark. Vendors of patent medicines and bottlers of all kinds will find these seals a great improvement on the old wax variety, and an absolute guarantee that the purchaser shall receive the goods as they left the manufacturers' hands.

